# Antioxidants in Alzheimer’s Disease: Current Therapeutic Significance and Future Prospects

**DOI:** 10.3390/biology11020212

**Published:** 2022-01-28

**Authors:** Pingal Pritam, Rahul Deka, Anuradha Bhardwaj, Rashi Srivastava, Dhruv Kumar, Abhimanyu Kumar Jha, Niraj Kumar Jha, Chiara Villa, Saurabh Kumar Jha

**Affiliations:** 1 Department of Biotechnology, School of Engineering and Technology, Sharda University, Greater Noida 201310, India; pingalpritam1@gmail.com (P.P.); rahuldeka.celtic@yahoo.com (R.D.); anu.consensus@gmail.com (A.B.); abhimanyu.kumar@sharda.ac.in (A.K.J.); niraj.jha@sharda.ac.in (N.K.J.); 2 Department of Chemical & Biochemical Engineering, Indian Institute of Technology, Patna 800013, India; rashisrivastava923@gmail.com; 3 Amity Institute of Molecular Medicine & Stem Cell Research, Amity University Uttar Pradesh, Noida 201313, India; dkumar13@amity.edu; 4 School of Medicine and Surgery, University of Milano-Bicocca, 20900 Monza, Italy; chiara.villa@unimib.it

**Keywords:** Alzheimer’s disease, antioxidants, oxidative stress, reactive oxygen species, therapeutics

## Abstract

**Simple Summary:**

Alzheimer’s disease (AD) is the most common neurodegenerative disease, intensifying impairments in cognition, behavior, and memory. Histopathological AD variations include extracellular senile plaques’ formation, tangling of intracellular neurofibrils, and synaptic and neuronal loss in the brain. Multiple evidence directly indicates that oxidative stress participates in an early phase of AD before cytopathology. Oxidative stress plays a crucial role in activating and causing various cell signaling pathways that result in lesion formations of toxic substances, which advances the disease. Antioxidants are widely preferred to combat oxidative stress, and those derived from natural sources, which are often incorporated into dietary habits, can play an important role in delaying the onset as well as reducing the progression of AD. However, this approach has not been extensively explored yet. Moreover, a combination of antioxidants in conjugation with a nutrient-rich diet might be more effective in tackling AD pathogenesis. Thus, considering the above-stated fact, this comprehensive review aims to elaborate the basics of AD and antioxidants, including the vitality of antioxidants in AD. Moreover, this review may help researchers to develop effectively and potentially improved antioxidant therapeutic strategies for this disease as it also deals with the clinical trials in the stated field.

**Abstract:**

Alzheimer’s disease (AD) rate is accelerating with the increasing aging of the world’s population. The World Health Organization (WHO) stated AD as a global health priority. According to the WHO report, around 82 million people in 2030 and 152 million in 2050 will develop dementia (AD contributes 60% to 70% of cases), considering the current scenario. AD is the most common neurodegenerative disease, intensifying impairments in cognition, behavior, and memory. Histopathological AD variations include extracellular senile plaques’ formation, tangling of intracellular neurofibrils, and synaptic and neuronal loss in the brain. Multiple evidence directly indicates that oxidative stress participates in an early phase of AD before cytopathology. Moreover, oxidative stress is induced by almost all misfolded protein lumps like α-synuclein, amyloid-β, and others. Oxidative stress plays a crucial role in activating and causing various cell signaling pathways that result in lesion formations of toxic substances, which foster the development of the disease. Antioxidants are widely preferred to combat oxidative stress, and those derived from natural sources, which are often incorporated into dietary habits, can play an important role in delaying the onset as well as reducing the progression of AD. However, this approach has not been extensively explored yet. Moreover, there has been growing evidence that a combination of antioxidants in conjugation with a nutrient-rich diet might be more effective in tackling AD pathogenesis. Thus, considering the above-stated fact, this comprehensive review aims to elaborate the basics of AD and antioxidants, including the vitality of antioxidants in AD. Moreover, this review may help researchers to develop effectively and potentially improved antioxidant therapeutic strategies for this disease as it also deals with the clinical trials in the stated field.

## 1. Introduction

**Figure 1:**
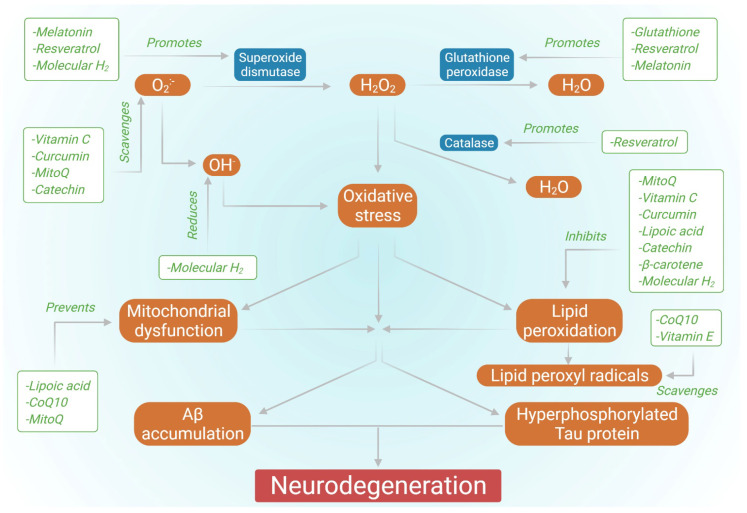
Effect of various antioxidants in countering AD.

According to the World Health Organization (WHO), around 50 million individuals worldwide suffer from dementia, with roughly 10 million new cases occurring each year [1]. Alzheimer’s disease (AD) is the most frequent cause of dementia, accounting for 60 percent to 70 percent of all cases [1]. AD is an irreversible, progressive, and accelerating brain disorder that results in loss of memory, thinking capacity, and, seemingly, the loss of ability to complete a simple task [2]. Although AD has a multifactorial etiology, it is characterized histopathologically by the presence of intracellular neurofibrillary tangles (NFTs) and extracellular senile plaques. NFTs are generated by the hyperphosphorylation of tau, a microtubule-associated protein localized to the axons and associated with the proper functioning of the cytoskeletal. When hyperphosphorylated in AD, tau protein leads to neuron dystrophy, aberrant skeletal framework, injury to axonal transport, and disrupted cell functions [3,4]. Senile plaques are composed of amyloid-β (Aβ) peptides of different lengths and are particularly resistant to degradation, resulting from the sequential proteolytic cleavage of Aβ precursor protein (APP) by β- and γ-secretases. The hydrophilic portion of the Aβ42 peptide coordinately forms a bond with transition metal ions such as copper (II), resulting in abnormal yet stable neurotoxic aggregates of Aβ [5]. Furthermore, Aβ has been shown to have the ability to disturb calcium homeostasis in neurons by activating calcium channels in the intracellular and plasma membrane of neurons [6]. Moreover, Aβ causes lipid peroxidation by interacting with the lipid membrane depending on the 4-HNE (4-hydroxynonenal) induced oxidation of cysteine residue, which leads to the interaction of this lipid peroxidation product with the membrane proteins specific to the brain and disrupts their structure and functionality. Such an escalation can be detrimental to the metabolic proteins of the brain. This activity of Aβ suggests the association between oxidative stress and Aβ deposition [7]. NFTs and senile plaques accelerate the neuroinflammatory responses, determine cytoskeletal stresses, and promote neuronal dysfunction [8,9,10]. Hence, one can easily infer that oxidative stress is crucial in inducing or activating the signaling pathways, leading to AD development. Although multiple approaches for the treatment of AD have been studied, at present, only a few drugs have been FDA approved for therapeutic applications (Table 1), so there is scope for novel therapeutic approaches in this regard. Several research studies have been conducted highlighting the effect of antioxidants in AD (Figure 1). Therefore, this review discusses the role of antioxidants and their related therapy for AD.

## 2. Oxidative Stress and Alzheimer’s Disease

Oxidative stress is defined as an imbalance between oxidants and antioxidants that causes a rise in oxidant levels [11,12,13,14]. It is well recognized as one of the clinical markers of AD; nevertheless, it is still unclear whether oxidative stress is a cause or a consequence of the process that occurs in AD patients’ brains. Reactive oxygen species (ROS) and Aβ are the major mediators of oxidative stress. The increase in the level of non-enzymatic glycation of cellular proteins, lipoproteins, and nucleic acids results from increased glucose levels [15]. These products are known as advanced glycation end products (AGEs) [16]. The administration of a diet rich in AGEs in the mouse hippocampus results in oxidative damage to the vasculature, increments in the level of Aβ, and memory impairments [17]. The receptor for AGEs is RAGE (receptor for advanced glycation end-products), a pattern recognition receptor that may bind massive ligands produced from the damaged cellular environment [18] and Aβ [19]. The NADPH oxidase (NOX) is activated by RAGE’s strong interactions with its ligands, resulting in increased ROS production [20]. *AGER* (gene encoding polymorphism in RAGE) has been linked to a hereditary predisposition to AD [17].

There are several pieces of evidence like APP23 mice carrying APP KM670/671NL mutation, which proves the early involvement of oxidative stress even before the deposition of Aβ in AD, [21], where triple transgenic mice carrying PS1 M146 V, Tau P301L, and APP KM670/671NL mutations were studied [22]. In the oxidative condition, Aβ generation may also be affected by the PS1/γ-secretase complex. It has been reported that 4-hydroxynonenal (4-HNE) or 4, 4-dithiodipyridine (DTDP) induces the pathogenic shift in the arrangement of PS1 subdomains within the γ-secretase complex resulting in enhancement of the aggregation and generation of Aβ species [23,24,25].

Furthermore, it has been demonstrated in recent research that not only may ROS regulate Aβ secretion/production, but that Aβ can also encourage the excessive development of ROS [26]. Overproduction of Aβ (as a result of APP overexpression) lowers the respiratory control ratio (RCR) and ATP production. It increases the production of ROS in HEK293 cells [27], implying the existence of a positive feedback loop between Aβ and ROS. ROS causes cPLA2 (calcium-dependent phospholipase A2) to be activated downstream, as well as phospholipid membrane disturbances, arachidonic acid release, and kinase activation [28,29,30], which are interlinked with the cause of AD.

The induction of oxidative stress by Aβ/APP has been reported in several studies [31]. For instance, APP KM670/671 NL and APP V717F mutation in the mouse model reported elevated lipid and protein oxidation markers like 3-NT (3-nitrotyrosine), 4-HNE, and others. Notably, the oxidative damage appears to become pronounced following the interaction of the sulfur-free radical with methionine 35 in the Aβ peptide [32,33].

## 3. Oxidative Stress Biomarkers in Blood Cells

Biomarkers detection helps in the early identification of AD. Brain imaging markers currently in use are neither cost-effective nor readily available [34]. However, blood-based markers can nail this as they are cost-effective, easily identifiable, and detectable. Moreover, detecting a blood-based biomarker can be repeated, and it is easily applicable in an aging population. Recently, depending upon oxidative stress, blood-based markers, most prominently cerebrospinal fluid (CSF) neurofilament light protein (NFL), plasma phospho tau (P-tau), total-tau (T-tau), and CSF Aβ42 are used to identify early AD [35]. Several pieces of the research reported that the imbalance in antioxidant defense and oxidative stress arising in the brain is reflected in the blood, which can further be easily accessed and used for early AD diagnosis [34,36,37,38]. In comparison to controls, ROS levels were higher in lymphocytes and platelets [39,40]. Protein carbonyls, 3-NT, NOS-2 (nitric oxide synthase 2), 4HNE, and other oxidative stress indicators appear to correlate with AD [41,42]. Furthermore, some investigations have explicitly linked oxidative stress to the early overproduction of Aβ [43,44,45]. Again, oxidative damage can be seen in proteins, lipids, and nucleic acids [46,47]. An increase in the levels of 8-hydroxy-2-deoxyguanosine (8-OHdG) and 8-hydroxyguanosine (8OHD), which are primarily localized in Aβ plaques, assesses oxidation in DNA and RNA [44]. Thus, these studies support the hypothesis that oxidative damage to lipids, nucleic acids, and proteins is a systematic series of events in peripheral cells in the blood.

## 4. Antioxidants

Antioxidants are compounds that can lower the harmful effects of oxidative stress. Antioxidants effectively decrease the rate of oxidation stress even in mild concentrations [48]. Based on the mechanism of action, antioxidants are mainly classified into two categories: (1) primary antioxidants and (2) secondary antioxidants. The former is responsible for scavenging free radicals and inhibiting the chain reaction resulting in oxidative stress. The latter undergo oxidation to decompose hydroperoxides into stable forms, thus exhibiting a synergistic effect with primary antioxidants. Secondary antioxidants are mainly involved in the regeneration of antioxidants, deactivation of metals, and reduction of singlet oxygen. As oxidative stress plays a vital role in AD likewise, antioxidants have been beneficial for AD [49]. An intricate natural antioxidant system in our body protects and prevents us from the damage caused by pro-oxidants [50]. Several reports show that various dietary sources can act as antioxidants [51,52,53]. Thus, the antioxidants system is classified into two types: (1) endogenous system and (2) exogenous system. Our body makes endogenous antioxidants, but humans procure exogenous antioxidants through diet. Glutathione peroxidase, catalase (CAT), glutathione reductase, superoxide dismutase (SOD), and others are directly involved in eliminating ROS. Nevertheless, cellular compounds like NADPH, vitamin C, mannitol, bilirubin, GSH, β-carotene, and others can be significant antioxidants.

Antioxidants with potential therapeutic applications against AD are shown in Table 2.

## 5. Role of Antioxidant-Rich Diet in Alzheimer’s Disease

It is well established that diet affects both mental and physical health. Food sources have a significant role in treating AD, as reported in many studies [88,89,90]. Vitamin C, E, carotenoids, flavonoids, polyphenols, and others are included in natural dietary antioxidants [91]. Moreover, it is suggested by literature and clinical reports that a proper diet including vitamins, proteins, and minerals will surely complement the medicine for the treatment of AD [89,90,91]. Apple cider has been reported to increase the activity of SOD, CAT, and glutathione peroxidase (GPx) to reduce lipid peroxidation [92,93].

**Figure 2:**
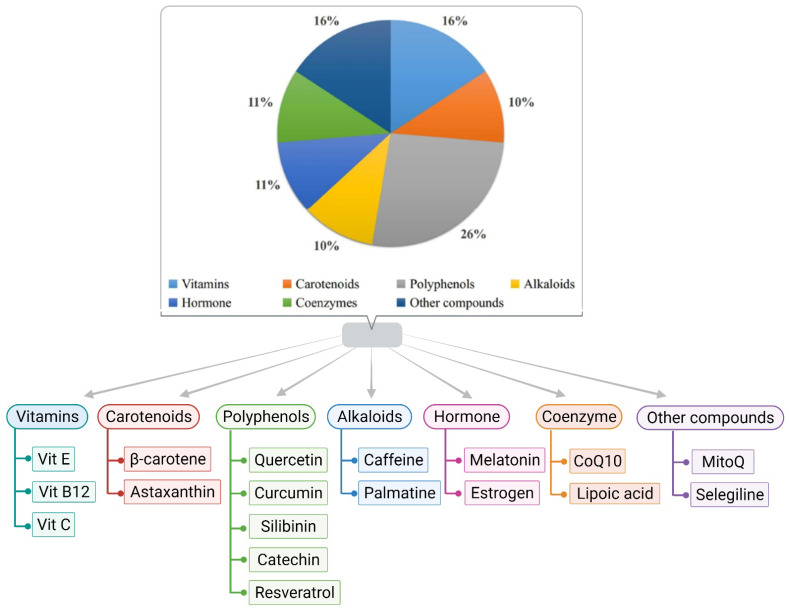
Various classes of antioxidants are documented for having the potential to counter AD.

Dietary potassium helps reduce ROS, alters the Aβ aggregation pattern, and helps in improving cognitive abilities [94]. Furthermore, increasing dietary potassium (to the optimum concentration) benefits individuals by preventing or delaying age and diet-related neurodegenerative diseases [95]. Garlic and its components show an admirable effect on brain function and neuronal physiology, leading to pharmacotherapy for AD [96]. Citrus fruits containing flavanone glycoside may be responsible for the conformational change of the beta-amyloid precursor protein cleaving enzyme 1 [97]. It has been found that supplementation of soaked almonds in the AD animal models of C57Bl/6 mice and adults of Sprague–Dawley rats, after overnight fasting, enhances memory due to the enrichment of vitamin E [97]. Despite under-conducted research, the cross-sectional studies of the overall role of diet and its patterns in AD are still questionable. Thus, research in this area is also needed. Moreover, people’s apprehension of the quantity and quality of individual bioactive components present in food items is insufficient for significant neuroprotection. Different classes of antioxidants with potential therapeutic applications against AD are displayed in Figure 2.

## 6. Role of Antioxidants in Alzheimer’s Disease

### 6.1. Vitamin E

Vitamin E is the most promising antioxidant for peroxyl radicals [98]. It can act on lipid-soluble membrane lipoproteins and low-density lipoproteins [99]. It has the potential to inhibit and delay neuronal death caused by inflammation. Moreover, it eliminates free radicals present in the red blood cell membrane and inhibits the spread of lipoperoxidation [100]. Furthermore, α-tocopherol is the most abundant form of vitamin E with high bioavailability in human tissue [101,102]. Vitamin E can be helpful in overcoming the increased expression of alpha-tocopherol transfer protein (α-TTP) in the patient’s brain suffering from AD [103]. A meta-analysis report of AD patients shows a reduced level of vitamin E in the blood plasma [104]. In one of the clinical trials, vitamin E and *Ginkgo biloba* extract were potentially significant in improving cognitive function of the brain [11]. Additionally, in another meta-analysis, it has been reported that a low concentration of serum vitamin E is associated with AD [105]. Moreover, substantial evidence suggested that vitamin E successfully suppresses tau-induced neurotoxicity in Drosophila [106,107,108]. In one of the recent studies, it is proposed that vitamin E has significantly reduced oxidative and nitrosative damage in AD [109]. However, the positive effect being evaluated of vitamin E in AD is still in the ongoing phases of various clinical trials.

### 6.2. Glutathione

Glutathione also plays a significant role in protein and DNA synthesis, cell cycle regulation, and storage and transport of cysteine. It has the potential to scavenge lipid peroxidation products like acrolein, 4-hydroxy-2-nonenal (HNE), and others [110]. It is used to maintain the thiol redox of cells, detox electrophiles, and metals, and protect from oxidative stress. It also can form metal complexes that reduce the toxicity of the metals and facilitate their further excretion from the body [110,111,112]. Recently, it was reviewed that cholesterol-mediated depletion of mitochondrial glutathione is linked with increased Aβ-induced oxidative stress in mitochondria [113]. The introduction of glutathione ethyl ester in transgenic mice featuring a high expression of sterol regulatory element-binding protein-2 (SREBP-2) has been shown to prevent neuroinflammation and neuronal damage [114]. Further, one recent study reveals the redox pathway of glutathione antioxidant responsible for regulating mitochondrial dynamics in axons [115]. However, the mechanistic overview of the exclusive role of glutathione in AD is still unclear.

### 6.3. Molecular Hydrogen

Molecular hydrogen is also an antioxidant that can modulate the Keap1-Nrf2-ARE signaling pathway and reduce inflammation [116]. It has a potential role in the selective reduction of hydroxyl radicals involved in the demolishing of proteins, nucleic acid and leads to lipid peroxidation, which is also a reported feature in AD [117]. It has been reported that molecular hydrogen administration increases short-lived Drosophila’s survival and life span [118]. At the same time, it is found that the hydrogen-rich water causes the increment in the level of glutathione and SOD [52]. Having both an indirect and direct role, the application of molecular hydrogen shows satisfying results for AD. However, more human trials are required for solid suggestions and recommendations.

### 6.4. Monoamine Oxidase-b Inhibitor

Monoamine oxidase catalyzes the oxidative deamination of xenobiotic and biogenic amines. In peripheral tissue and the central nervous system, they play an important role in the metabolism and control of vasoactive and neuroactive amines. In cerebral blood arteries, a monoamine oxidase-b inhibitor can rapidly produce the vasodilator nitric oxide [119]. By blocking oxidative deamination, it shields the vascular endothelium from the effects of Aβ and improves the survival and function of nigral neurons [120,121]. It is also reported to decrease the progression of AD by reducing neuronal damage [122]. L-deprenyl, a monoamine oxidase-b inhibitor, enhances nitric oxide production accompanied by vasodilation; however, the study also suggests that L-deprenyl may involve other pathways for its effectivity [119].

### 6.5. Melatonin

Melatonin, a mammalian hormone synthesized in the pineal gland, can scavenge oxygen and nitrogen-based reactants. It performs by stimulating and promoting the activity and expression of NO synthase, SOD, and GPx [123]. It has a significant role in reducing oxidative damage of cells [124]. In recent literature, it has been reported that antioxidant melatonin can mitigate tau hyperphosphorylation [125,126,127,128,129] and inhibit the toxicity induced by Aβ [130].

### 6.6. Ascorbyl Palmitate

It is a lipid-soluble form of vitamin C. It maintains all the vitamin C activity without creating problems associated with ascorbic acids, such as less recycling capacity of α-tocopherol in the lipid bilayer, reduced viability in-vivo, and others [122]. Additionally, it is reported that the demand for vitamin C can be better fulfilled with lipophilic form rather than hydrophilic form [131]. Ascorbyl palmitate can successfully cross the blood-brain barrier (BBB) [132] and is reported for its significant role in treating AD [133]. As ascorbyl palmitate resides in the cell membrane, it can accelerate the production of vitamin E. However, the protective role of vitamin C is still in debate as it is not yet clear whether vitamin C is acting alone or in combination for treating AD.

### 6.7. Curcumin

Multiple desirable features reside in curcumin for a neuroprotective drug, including antioxidant, anti-protein aggregates, and anti-inflammatory activities [134]. It has been studied that curcumin reduces inflammation, oxidative damage, and cognitive deficits in rats where Aβ toxicity has affected their central nervous system. Curcumin possesses substantial free radical scavenging properties, whereby it targets NO-based radicals to scavenge them, which helps inhibit lipid peroxidation [135]. Curcumin has also been reported to bind with metal ions, which prevents them from causing aggregation of Aβ and reduces oxidative stress [136]. Moreover, curcumin was also found to restore glutathione levels in brain tissue and reduce oxidized proteins in mice models with AD [137,138]. However, in one of the clinical trials, curcumin’s beneficial effect in AD couldn’t be determined; this may be due to highly poor pharmacokinetics and pharmacodynamics properties [139].

### 6.8. Coenzyme Q and SK-PC-B70M

Coenzyme Q is currently studied for its role in Parkinson’s disease and amyotrophic lateral sclerosis [106]. Moreover, it helps in the generation of ATP. It is the only lipid synthesized directly within the body and can maintain a redox function [140]. Coenzyme Q has the potential to neutralize free radicals and stabilize the optimal functioning of the cell membrane. The contribution of coenzyme Q in AD treatment must be explored as there is a high possibility that it might play an influential, protective, and preventing role in AD. SK-PC-B70M, an oleanolic-glycoside saponin enriched fraction, is derived from Pulsatilla Korean. Currently, it has been reported for its neuroprotective activity against the cytotoxicity effect induced by Aβ in SK-N-SH [141].

### 6.9. Estrogen, Astaxanthin, and Quercetin

Estrogen protects neurons against the toxicity of Aβ by acting as an antioxidant [142]. It appears to have a neuroprotective effect [52] without improving function or cognition in people with AD [142]. Astaxanthin is a powerful carotenoid that can prevent apoptosis, oxidative stress, inflammation, memory loss, and protect against Aβ’s neurotoxic effects [94,142,143,144]. Quercetin is the most prominent and significant dietary antioxidant effective on health as it protects against severe diseases like lung cancer, cardiovascular disease, osteoporosis, and others [145]. There are ongoing clinical trials for estimating its accurate effect on AD [146].

### 6.10. Lipoic Acid

The medicinal antioxidant lipoic acid (α-lipoic acid) is found in the mitochondria. Pyruvate dehydrogenase and α-ketoglutarate dehydrogenase both use it as a cofactor. However, it is also involved in the recycling of other antioxidants such as vitamin C and E, as well as glutathione, in order to boost ACh production [53]. Lipoic acid is also implicated in some redox-active chelating metals, which helps to prevent lipid peroxidation from building up [147]. When used in combination with acetylcarnitine, lipoic acid was found to protect neuronal cells through cell-signaling pathways, including specific extracellular kinase pathways, mainly the Ras-MAPK pathway that were dysregulated in AD [148]. Studies undertaken on the brain of control and AD mouse models showed that lipoic acid reduced the expression of F2 isoprostanes and neuroprostanes, which are oxidative stress markers [149]. Lipoic acid also induces the transcription factor Nrf2, which regulates a number of different antioxidant enzymes involved in protection from oxidative stress [150]. Lipoic acid improved memory and reversed oxidative stress indices in the senescence-accelerated mouse-prone 8 (SAMP8) models [151]. Lipoic acid is a potent antioxidant as it can traverse the BBB, making it ideal for therapeutic applications in AD [152].

### 6.11. Resveratrol

Resveratrol (3, 5, 4′-trihydroxy-trans-stilbene) is a polyphenolic compound found in a number of plants, like red grapes, blueberries, dark chocolate, and peanut butter. Resveratrol has been reported to possess antioxidant properties and was found to diminish malondialdehyde and nitrite levels and restore glutathione levels [84]. Studies in a number of cell lines expressing mutant AβPP695 reported that resveratrol exhibited anti-amyloidogenic activity through reduction in secreted intracellular Aβ peptide levels [153]. Levels of intracellular antioxidant enzymes SOD, CAT, GPx, and HO-1 were increased by resveratrol while simultaneously reducing lipid peroxidation [79]. Another essential function of resveratrol was diminishing ROS production in brain tissue by preventing disruption in the mitochondrial membrane potential [154]. The binding of metal ions to Aβ and NFTs enhances their aggregation and increases ROS production. Resveratrol counteracts this through dysregulation of the metal ion balance [84]. Along with antioxidant properties, resveratrol has been reported to promote an anti-inflammatory response, reduce levels of tau protein phosphorylation and increase the activity of SIRT-1 [154]. This makes resveratrol an interesting natural antioxidant in combating AD pathogenesis.

### 6.12. MitoQ

MitoQ is an antioxidant that targets the mitochondria in AD. MitoQ is made by adding the lipophilic triphenylphosphonium (TPP^+^) cation to ubiquinone, a component of the mitochondrial electron transport chain, via a ten-carbon chain [155]. TPP^+^ facilitates entry of ubiquinone into the mitochondrial matrix, where the complex II reduces ubiquinone to ubiquinol, the active antioxidant form, decreasing lipid peroxidation, which reduces oxidative damage [156]. MitoQ is able to traverse the BBB rapidly and has been found to accumulate several hundred folds in the mitochondrial membrane. The uptake of MitoQ in the mitochondria is driven by the high membrane potential of the inner mitochondrial membrane [157]. MitoQ has been found to reduce free radicals and oxidative damage while helping to regulate mitochondrial functions of the cells [158]. MitoQ was found to lower Aβ peptide levels, minimize synaptic loss and astrogliosis and improve cognitive functions in AD mouse model studies wherein the administration of MitoQ was initiated at a young age [155,159]. MitoQ was also reported to enhance neurite outgrowth in neurons and protection against Aβ peptide toxicity in cells of AD mouse models [160].

### 6.13. Catechins

Catechins are the bioactive components found in tea—most abundant in green tea (green tea catechins or GTC)—which includes four different types of catechins: viz. epicatechin (EC), epicatechin gallate (ECG), epigallocatechin (EGC), and epigallocatechin gallate (EGCG) [153]. Catechins exhibit antioxidative effects by scavenging ROS and chelating metal ions like copper, iron, and zinc, thereby reducing their accumulation in the brain of AD patients [86]. EGCG was reported to reduce caspase levels and oxidative stress along with reducing lipid peroxidation in the hippocampus of the rat model [161]. A long-term study on male Wistar rats revealed that the administration of 0.5% GTC in water resulted in counteracting Aβ-induced cognitive impairment, along with reduced levels of plasma lipid peroxide and ROS levels [162]. In addition toantioxidant properties, catechins were also reported to exhibit anti-inflammatory properties, along with inhibition of acetylcholinesterase (AChE) activity. At the same time, EGCG was found to directly interact with Aβ peptides and prevent the formation of aggregates [86,163,164]. Furthermore, catechins are BBB permeable, as found in rodent models, making them a potential therapeutic candidate for AD treatment [86].

### 6.14. Silibinin

Silibinin, an antioxidant flavonolignan obtained from *Silybum marianum*, can boost the amount of newly formed microglia, astrocytes, neurons, and neural precursor cells in the brain [165]. In one study, silibinin was found to be a dual inhibitor of AChE and Aβ peptide aggregation, implying a therapeutic method for treating Alzheimer’s disease [165]. It can potentially prevent the injuries caused by Aβ1-42-indued oxidative stress by lowering the production of H_2_O_2_ in Aβ1-42-stressed neurons [166]. Another study reported that streptozotocin-induced tau hyperphosphorylation (ser404) in the hippocampus was substantially reduced by silibinin [167]. Though these results indicate that silibinin may be a novel therapeutic agent for treating AD, no clinical trials are on board.

### 6.15. Palmatine

Palmatine, an isoquinoline alkaloid, acts against Aβ induced neurotoxicity [168]. It is reported that palmatine activated the Nfr2 knockdown and AMPK pathway [168]. It is reported for having anti-inflammatory, antioxidative and antiproliferative effects [169]. Another study reported the combined impact of palmatine and berberine on the inhibition of AChE [169,170]. Though it is reported in several in-silico and in-vivo studies, there is still a massive absence of its proper application in AD. Moreover, the mode of action underlying their neuroprotective effect is poorly characterized in vivo.

### 6.16. Serotonin

Serotonin, an indoleamine neurotransmitter, can disassemble performed Aβ fibrils [171]. Ample evidence reflects that a combination of disturbances in serotonergic and cholinergic function may possess a vital role in cognitive impairment in AD [172]. In one study, it is indicated that alterations of the serotonergic system contribute to neuropsychiatric symptoms in AD as their results suggest that a decline in neurons expressing 5-HT2A plays a role in the etiopathology of neuropsychiatric symptoms in AD [173]. Furthermore, while many of these compounds will likely be used as adjuvant therapy in the treatment of AD symptoms, there are currently just a few pharmacological entities with activity against serotonin receptors that have the potential to slow the illness’s progression.

### 6.17. Gintonin

Gintonin, a glycol-lipoprotein, can help in maintaining the integrity of BBB [174]. It can suppress the activated inflammatory mediators and microglial cells in the brains of *A*β-injected mice [175]. Recent findings suggest that treatment with gintonin in AD results in improved synaptic and memory functions in the brain [176]. It reflects an emerging role as a modulator of neurogenesis and synaptic transmission, and it has the potential to regulate autophagy in primary cortical astrocytes [176,177]. Moreover, as a novel agonist of lysophosphatidic acid receptors, gintonin regulated several GPCR, including GPR55 and GPR40 [177]. Nevertheless, further exploration is still required to understand gintonin’s underlying mode of action in AD.

## 7. Role of Other Nutrients in Alzheimer’s Disease

Apart from antioxidant activities, natural products have exhibited other vital properties to combat AD progression through anti-inflammatory response, prevention of Aβ aggregation, accumulation of tau protein, and the promotion of cholinergic signaling [178]. Alkaloids, such as cryptolepine and tetrandrine, have been reported to be involved in the inhibition of NF-κB, thereby acting as anti-inflammatory agents [179]. Flavonoids, owing to their characteristic property of inhibiting inflammatory response, have shown potential for working against AD progression [180]. Studies in animal models of AD have reported terpenoids, such as artemisinin, parthenolide, and carnosol can inhibit NF-κB and p38 MAPK pathways [181,182,183]. Ginsenoside Rg1, a compound obtained from the roots of the Ginseng plant, has been reported to cause a significant drop in levels of Aβ peptide levels in AD mice [184]. Natural plant products like crocin, α-cyperone, chrysophanol, and aloe-emodin have been found to exhibit properties that inhibit tau protein formation and reduce AD progression [185,186,187]. Caffeine, one of the most widely consumed alkaloids, has been found to inhibit Aβ deposition in vitro [188]. It was also found to reduce ROS production and enhance SOD levels in human neuroblastoma cells cultured with Aβ [189]. Caffeine has also been shown to exhibit anti-neuroinflammatory properties as well as decreasing tau protein phosphorylation in the hippocampus [190]. In low to moderate doses, caffeine inhibits AChE, thereby improving cognitive actions and reducing the progression of AD [191]. Eugenol, found in cloves, has been reported to reduce amyloid plagues and increase memory in rat models induced with Aβ peptides [192]. Dietary patterns have also been found to impact the onset and progression of AD. A Western diet characterized by higher meat intake was associated with an increased risk of AD [193]. In contrast to this, the Mediterranean diet, characterized by higher consumption of fruits, vegetables, and fish with lower meat intake, was found to reduce the risk of AD in the population [194].

## 8. Limitations and Future Perspectives

Antioxidants have been shown to be effective against AD. The clinical trials for evaluating the therapeutic potential for several antioxidants in AD are shown in Table 3. But the availability of data related to pre-clinical studies falls short to justify their widespread application [195]. Antioxidants have been shown to lower the damage by oxidative stress in the brain though limited human trials and make it difficult to conclude accordingly. The research interventions for AD should mainly focus on the patient’s lifestyle, keeping in mind their cognitive status. The application of antioxidants and their effect could be monitored in AD patients through the nutrient-rich diet they are being advised for intake [196]. A holistic approach towards the treatment of AD has become the need of the hour. AD treatment needs to be addressed at the right time to minimize the chances of failure. Genomic sequencing commenced by the National Institutes of Health (NIH) in 2012 has opened up new avenues in developing a more contemporary and specialized treatment for AD [197]. Inflammatory responses have been an innate part of AD pathogenesis that needs to be addressed with much-advanced technology as soon as possible. ROS poses a significant threat to neurons. Oxidative damage is a prominent pathological symbol for AD. Antioxidants have ROS scavenging ability, making them a feasible candidate in the fight against AD [198]. Nonetheless, inconclusive results from human trials make it difficult for the physician to recommend AD treatment. The synergistic administration of various antioxidants could counter oxidative stress with much efficiency. The concentration of antioxidants administered should be taken care of since high doses could disrupt the normal physiological process where ROS plays a prominent role. Extensive studies should be carried upon placing a closer look at toxicity, bioavailability issues, and long-term exposure of antioxidants in AD patients [199]. The most important aspects to look for while carrying out antioxidant-based research for AD are the time-span of consumption and the age group of people above 60 for the administration of antioxidants. The onset of dementia might get initiated even before the appearance of definitive symptoms [200]. The requirement of biomarker identification and neuropathological assessment has become a must for diagnosing and providing the antioxidant at the required time-span before the onset of AD [201]. Limitations of antioxidants, such as lower bioavailability of polyphenols, resveratrol, and others require proper research interventions [202]. Mixing polyphenols with juice or extracts from fruits could be looked for extensively, in order to mediate the shortcomings of polyphenol therapy for AD [203]. The viable method of administering antioxidants needs to be addressed with further pharmacological studies since some patients may not be comfortable with tablets, and some may not be with extracts. Thus, the viable mode needs to be addressed according to the patient’s needs [204]. Additionally, the intervention of nanotechnology could be crucial towards the enhancement of bioavailability and moving past the BBB, which is another shortcoming of antioxidants being administered conventionally [205]. The role of antioxidants could be vital in the battle against a plethora of neurodegenerative diseases. Therefore, extensive human trials are required to test the efficacy of the antioxidant in AD patients.

## 9. Conclusions

With a long asymptomatic period, AD is a chronic neurodegenerative condition. Multiple literature and evidence infer that oxidative damage or stress plays a significant role in the pathogenesis of AD through various mechanisms and pathways. Thus, new treatment strategies are required to either prevent or reduce oxidative damage and may provide therapeutic efficacy against AD. Natural bioactive, often incorporated into the diet, can become a widely adopted approach to avoid the onset of AD. Moreover, this approach can be conjugated with approved drugs for patients with progressive AD. The integrated system of antioxidants with multiple drugs may provide higher effectiveness. Some antioxidants have proven positive effectors on AD, but some still need attention and work. Moreover, there is limited data on the role of antioxidants in AD from human clinical trials and epidemiological studies. Additionally, some antioxidants show significant effects on an animal model but exhibit diminished efficacy on humans during clinical trials. Due to this, there is a lot of skepticism about the success of antioxidant therapy for AD. It is quite necessary to explore a more definitive and precise approach integrated with antioxidants for lowering or inhibiting the progression of AD. The link between inflammation and AD is unavoidable, so antioxidants’ integrated role in decreasing inflammation must be considered. Thus, further advanced studies and human clinical trials are necessary to determine and estimate the antioxidants potential for AD.

## Figures and Tables

**Table 1:** FDA approved drugs for the treatment of AD (Adapted from Medications for Memory Loss|Alzheimer’s Association).

Drug Name (Generic/Brand)	Manufacturer	Drug Type	Drug Use	Mechanism	Side Effects
Aducanumab Aduhelm™	Biogen, Eisai Co. Ltd., Tokyo, Japan	Disease-modifying immunotherapy	Alzheimer’s disease (MCI or mild dementia)	Removes abnormal Aβ helping to reduce the number of plaques in the brain	Amyloid-related imaging abnormalities (ARIA), which can lead to fluid buildup or bleeding in the brain; headache, dizziness, falls, diarrhea, confusion
Donepezil Aricept^®^	Eisai Inc. and Pfizer Inc., New York, NY, USA	Cholinesterase inhibitor	Mild, moderate, and severe symptoms of AD	Prevents the breakdown of acetylcholine in the brain	Nausea, vomiting, diarrhea, muscle cramps, fatigue, weight loss, loss of appetite, and increased frequency of bowel movements.
Rivastigmine Exelon^®^	Novartis, Basel, Switzerland	Cholinesterase inhibitor	Mild to moderate symptoms of AD	Prevents acetylcholine and butyrylcholine from being degraded in the brain	Nausea, vomiting, diarrhea, weight loss, indigestion, muscle weakness
Galantamine Razadyne^®^	Ortho-McNeil Neurologics, Johnson & Johnson, Titusville, NJ, USA.	Cholinesterase inhibitor	Mild to moderate symptoms of AD	Prevents the breakdown of acetylcholine and stimulates nicotinic receptors to release more acetylcholine into the brain	Nausea, vomiting, diarrhea, decreased appetite, dizziness, headache
Memantine Namenda^®^	Allergan plc, Dublin, Ireland	N-methyl D-aspartate (NMDA) antagonist	Moderate to severe symptoms of AD	Blocks the toxic effects associated with excess glutamate and regulates glutamate activation	Dizziness, headache, diarrhea, constipation, confusion
Memantine + Donepezil Namzaric^®^	Actavis and Adamas Pharmaceuticals, Dublin, Ireland	NMDA antagonist and cholinesterase inhibitor	Moderate to severe symptoms of AD	Blocks the toxic effects associated with excess glutamate and prevents the breakdown of acetylcholine in the brain	Nausea, vomiting, loss of appetite, increased frequency of bowel movements, headache, constipation, confusion, and dizziness

**Table 2:** List of antioxidants with potential therapeutic effects against AD.

Antioxidant	Chemical Structure	Functions	References
Vitamin E	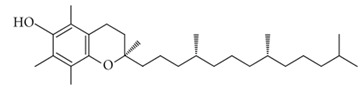	Lowers down free radical-mediated neuronal toxicity Inhibits dementia pathogenesis in mammalian culture cells	[54,55]
Vitamin C	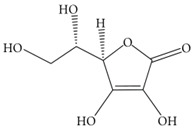	Inhibits lipid peroxidation As a major defense barrier against free radicals in plasma cells	[56,57]
β carotene	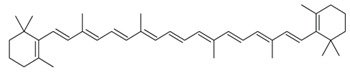	Reduces lipid peroxidation Helps in improving the status of antioxidant availability	[58,59]
Vitamin B12	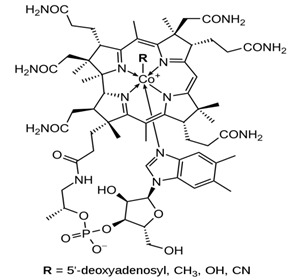	Increases choline acetyltransferase activity as depicted in cat model Helps in the improvement of cognitive ability in AD patients	[60,61]
α-lipoic acid	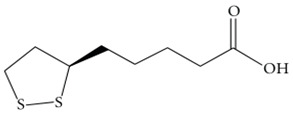	Helps in the recycling of other antioxidants such as Vitamin C, E, glutathione Increases the production of acetylcholine to counter reactive products arising from lipid peroxidation reactions.	[62,63]
CoQ10	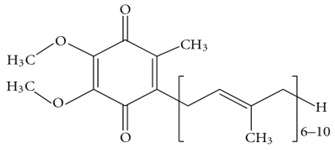	Protects mitochondrial membrane potential during oxidative stress condition Helps in neuronal protection against Aβ plaque accumulation	[64,65]
Caffeine	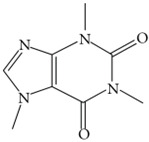	Inhibits amyloidosis and Aβ accumulation Reduces Aβ plaque levels in AD-associated mouse models	[66,67]
Curcumin	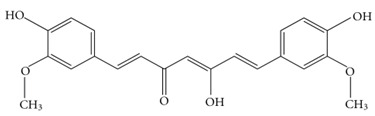	Inhibits enzymes such as lipoxygenase and cyclooxygenase 2 responsible for the synthesis of pro-inflammatory leukotrienes, prostaglandins, and thromboxanes Reduces Aβ plaque levels in AD-associated mouse models	[68,69]
Berberine	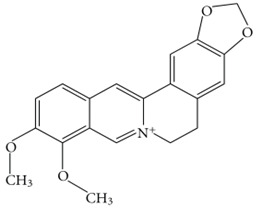	Reduces Aβ plaque levels Inhibits the formation of ROS and RNS	[69,70]
Palmatine	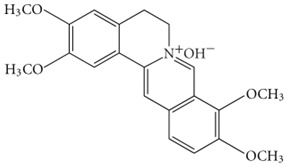	Reduces Aβ plaque levels Inhibits formation of ROS and RNS	[71,72]
Silibinin	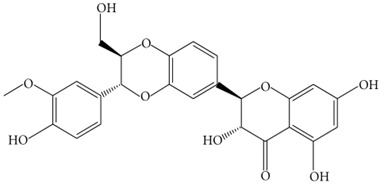	Prevents Aβ-mediated oxidative stress in mice models Prevents memory impairment in mice models	[73,74]
Quercetin	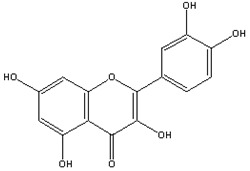	Inhibits Aβ aggregation in vitro Reduces the expression of APP	[75,76]
Melatonin	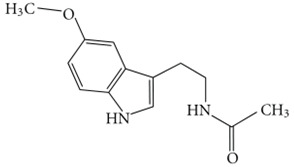	Helps in scavenging RNS generated in mitochondria by stimulating the expression of GPx, SOD, and NO synthase Reduces oxidative stress in mammalian cells	[77,78]
Estrogen	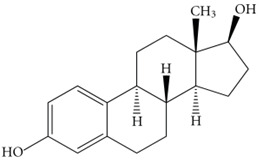	Helps in protection of neurons from the toxicity of Aβ	[78,79,80,81]
Selegiline	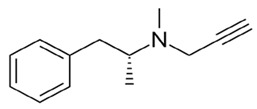	Helps in the protection of vascular endothelium from Aβ toxicity Helps in the protection of nigral neurons from oxidative deamination	[82,83]
Resveratrol	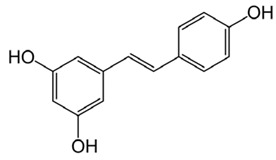	Helps in decreasing the production of Aβ peptides in vitro Helps in the improvement of memory deficit and cognitive impairment in experimental animal models Reduces the level of MDA in an animal model Protects cells from Aβ-induced toxicity in vitro	[84,85]
Tea polyphenols-(−)- epicatechin (EC) (−)-epicatechin-3-gallate (ECG) (−)-epigallocatechin (EGC) (−) epigallocatechin-3- gallate (EGCG)	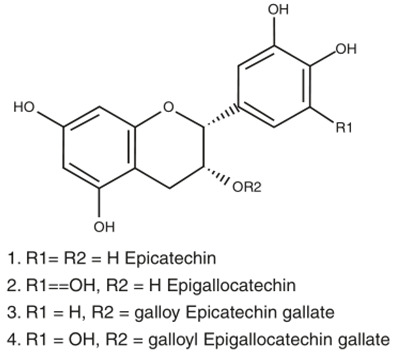	Helps in decreasing preformed Aβ fibrils in vitro Inhibits the self-aggregation of Aβ Reduces the production of Aβ peptides in AβPP695 overexpressing neurons Helps in reducing Aβ production in transgenic AD mice Reduces Aβ-induced neuronal cell death permeable to BBB Reduces Aβ-induced caspase activity in hippocampal neuronal cells	[86,87]

Aβ, amyloid-β; AD, Alzheimer’s disease; BBB, blood-brain barrier; GPx, glutathione peroxidase; RNS, reactive nitrogen species; ROS, reactive oxygen species; SOD, superoxide dismutase.

**Table 3:** Completed clinical trials conducted for antioxidants relevant to AD (“Completed” status here means the study has ended, and participants are no longer being examined or treated (that is, the last participant’s last visit has occurred).

Sl. No.	NCT Number	Conditions	Interventions	Outcome Measures	Phases
**1**	NCT00117403 (https://clinicaltrials.gov/show/NCT00117403, accessed on 20 December 2021)	AD	Drugs: Vitamin E, Vitamin C, and Alpha-lipoic Acid Drug: Coenzyme Q Drug: Placebo capsules Drug: Placebo wafers	Effect on CSF biomarkers related to oxidative damage change in plasma and CSF concentrations of Aβ42 and Aβ40	Phase 1
**2**	NCT00090402 (https://clinicaltrials.gov/show/NCT00090402, accessed on 20 December 2021)	AD Oxidative Stress Dementia Hyperlipidemia Inflammation	Dietary Supplement: Fish Oil Dietary Supplement: Lipoic Acid Other: Fish Oil Placebo Other: Lipoic Acid Placebo	F2-isoprostane Level: Urine F2-Isoprostanes Change in Mini-Mental State Exam (MMSE) Score From Baseline to 12 Months Change in Activities of Daily Living/Instrumental Activities of Daily Living (ADL/IADL) Scores From Baseline to 12 Months	Phase 1 Phase 2
**3**	NCT00678431 (https://clinicaltrials.gov/show/NCT00678431, accessed on 20 December 2021)	AD	Dietary Supplement: Resveratrol with Glucose and Malate Dietary Supplement: Placebo	Alzheimer’s Disease Assessment Scale (ADAScog) CGIC	Phase 3
**4**	NCT00000173 (https://clinicaltrials.gov/show/NCT00000173, accessed on 20 December 2021)	AD	Drug: Donepezil Drug: Vitamin E		Phase 3
**5**	NCT00951834 (https://clinicaltrials.gov/show/NCT00951834, accessed on 20 December 2021)	AD	Drug: Epigallocatechin-Gallate Drug: Placebo	ADAS-COG (Score 0–70) (Baseline to treatment) Safety and tolerability of the verum MMSE (Score 0–30) after 18 months compared to baseline Time to hospitalization and Time to death related to AD Brain atrophy assessed by brain MRI Baseline-ADAS-COG and Baseline-MMSE as covariates CIBIC+ and WHO-QOL-Bref Trail Making Test and MVGT	Phase 2 Phase 3
**6**	NCT01707719 (https://clinicaltrials.gov/show/NCT01707719, accessed on 20 December 2021)	AD Oxidative-Stress Adrenocortical-hyperfunction		Malondialdehyde assay Relationship between urinary excretion of cortisol and levels of malondialdehyde	
**7**	NCT00628017 (https://clinicaltrials.gov/show/NCT00628017, accessed on 20 December 2021)	AD Mild Cognitive Impairment	Dietary Supplement: omega-3 polyunsaturated fatty acids (EPA+DHA)	The Clinician’s Interview-Based Impression of Change Scale (CIBIC-plus) The cognitive portion of the Alzheimer’s Disease Assessment Scale (ADAS-cog) Mini-Mental Status Examination (MMSE) score 17-item Hamilton Depression Scale (HDRS) 18-adverse events	Not Applicable
**8**	NCT00099710 (https://clinicaltrials.gov/show/NCT00099710, accessed on 20 December 2021)	AD	Dietary Supplement: Curcumin C3 Complex	Side effect checklist Oxidative damage Inflammation/gliosis A-beta levels Tau levels Total plasma cholesterol, LDL and HDL; ApoE Plasma curcumin and metabolites Cognitive and behavioral measures	Phase 2
**9**	NCT00597376 (https://clinicaltrials.gov/show/NCT00597376, accessed on 20 December 2021)	Subjective Memory Loss in Older Persons	Other: Cerefolin NAC (a medical food) Other: Cerefolin NAC placebo	Six-month blood levels of Homocysteine, Glutathione, and the Ratio of Aβ42 to Aβ40 (as a Percent of Baseline Levels) After Daily Intake of Cerefolin NAC Plus a Multivitamin Versus a Multivitamin Only Tolerability of Cerefolin NAC and a Multivitamin Versus a Multivitamin Only Six Month Levels of Inflammation and Oxidative Stress Markers (as a Percent of Baseline Levels) After Daily Treatment with Cerefolin NAC and a Multivitamin or a Multivitamin Only	Not Applicable
**10**	NCT00940589 (https://clinicaltrials.gov/show/NCT00940589, accessed on 20 December 2021)	AD Sleep Disorder	Drug: Circadin Drug: Placebo	Change From Baseline to 24 Weeks in ADAS-cog Change From Baseline to 24 Weeks in iADL Change From Baseline to 24 Weeks in MMSE	Phase 2
**11**	NCT00000171 (https://clinicaltrials.gov/show/NCT00000171, accessed on 20 December 2021)	AD Dyssomnias	Drug: Melatonin		Phase 3
**12**	NCT01058941 (https://clinicaltrials.gov/show/NCT01058941, accessed on 20 December 2021)	AD	Drug: Lipoic acid and fish oil concentrate Drug: Placebo	Change From Baseline in Activities of Daily Living (ADL) at 18 Months Change From Baseline in Alzheimer’s Disease Assessment Scale—Cognitive Subscale (ADAS-cog) at 18 Months	Phase 1 Phase 2
**13**	NCT01370954 (https://clinicaltrials.gov/show/NCT01370954, accessed on 20 December 2021)	Early Memory Loss MCI AD VaD	Other: CerefolinNAC^®^	To determine if CerefolinNAC^®^ affects a subject’s quality of life as measured by the Quality of Life-Alzheimer’s Disease Scale (QOL-AD) To determine overall patient satisfaction with CerefolinNAC^®^ using a 9-point satisfaction scale	
**14**	NCT01504854 (https://clinicaltrials.gov/show/NCT01504854, accessed on 20 December 2021)	AD	Drug: Resveratrol Drug: Placebo	Number of Adverse Events Change From Baseline in Volumetric Magnetic Resonance Imaging (MRI) Change in Alzheimer’s Disease Cooperative Study-Activities of Daily Living (ADCS-ADL) Comparison of the Response to Treatment of Resveratrol Based on ApoE Genotype	Phase 2
**15**	NCT00040378 (https://clinicaltrials.gov/show/NCT00040378, accessed on 20 December 2021)	AD	Drug: alphatocopherol Drug: Selenium Drug: Placebo replacement for vitamin E Drug: Placebo replacement for Selenium	Incidence of dementia (including Alzheimer’s disease)	
**16**	NCT00235716 (https://clinicaltrials.gov/show/NCT00235716, accessed on 20 December 2021)	AD	Drug: dl-alpha-tocopherol Drug: Memantine Drug: Placebo	Alzheimer’s Disease Cooperative Study/Activities of Daily Living (ADCS/ADL) Inventory Change From Baseline Mini-Mental State Examination Change From Baseline Alzheimer’s Disease Assessment Scale—Cognitive (ADAS-cog) Change From Baseline Neuropsychiatric Inventory Change From Baseline Caregiver Activity Survey Change From Baseline Dependence Scale: Time to Event Analysis (Increase of One Dependence Level)	Phase 3
**17**	NCT01716637 (https://clinicaltrials.gov/show/NCT01716637, accessed on 20 December 2021)	AD	Biological: Etanercept Dietary Supplement: Curcum.Luteol.Theaflav.Lip.Acid, FishOil, Quercet., Resveratr.	The difference in effects of treatment for 6 weeks with etanercept + nutritional supplements versus nutritional supplements alone on the Mini-Mental Status Examination (MMSE) score; The difference in the effects of treatment for six weeks with etanercept + nutritional supplements versus nutritional supplements alone on the Alzheimer’s Disease Assessment Scale-Cognitive Subscale (ADAS-cog) score; The difference in the effects of treatment for six weeks with etanercept + nutritional supplements versus nutritional supplements alone on the Montreal Cognitive Assessment (MoCA) score	Phase 1
**18**	NCT00692510 (https://clinicaltrials.gov/show/NCT00692510, accessed on 20 December 2021)	AD	Drug: AZD3480 Drug: Placebo Drug: Cocktail mix (Caffeine, Bupropion, Rosiglitazone, Omeprazole, Midazolam, Bilirubin)	PK variables Safety variables (adverse events, blood pressure, pulse, safety lab)	Phase 1
**19**	NCT01594346 (https://clinicaltrials.gov/show/NCT01594346, accessed on 20 December 2021)	AD DS	Drug: Alpha-Tocopherol Drug: Sugar Pill	The Brief Praxis Test The Fuld Object Memory Test New Dot Test Orientation Test Vocabulary Test Behavior and Function Clinical Global Impression Incident Dementia	Phase 3
**20**	NCT01780974 (https://clinicaltrials.gov/show/NCT01780974, accessed on 20 December 2021)	Treated Hypertension	Drug: Lipoic Acid plus Omega-3 Fatty Acids Drug: Placebo	Trails Making Test Part B (Executive Function) White Matter Hyperintensity Volume (Brain MRI)	Phase 1 Phase 2
**21**	NCT01699711 (https://clinicaltrials.gov/show/NCT01699711, accessed on 20 December 2021)	DS	Dietary Supplement: Epigallocatechin-3-gallate (EGCG)	Change in Cognitive Evaluation and Amyloidosis Biomarker Treatment compliance Change in Biomarkers of lipid oxidation and DYRK1A activity biomarkers COMT val158met genetic polymorphism (catechol methyl transferase) (Taqman) Change in AST (SGOT-serum glutamic oxaloacetic transaminase-) and ALT (SGPT-Serum Glutamic Pyruvate Transaminase-) (Pentra Autoanalyzer, and ELISA Mercodia for LDLox) Change in Body Composition by electrical impedance (TANITA-MC-180) Changes in Neurophysiology	Phase 2

Aβ, amyloid-β; Acetylcholinesterase, AChE; AD, Alzheimer’s disease; APOE, apolipoprotein E; CSF, cerebrospinal fluid; DS, Down syndrome; MMSE, mini-mental state examination; VaD, vascular dementia; MCI, Mild Cognitive Impairment.

## Data Availability

Not applicable.
